# Massive uterine angiomyolipoma mimicking uterine leiomyoma in a 49-year-old woman

**DOI:** 10.5339/qmj.2026.66

**Published:** 2026-09-08

**Authors:** Sheela Manikyam, Thendral Kalaimani, Ashmitaa Srianand, Femela Muniraj

**Affiliations:** 1 Department of Obstetrics and Gynecology, Kauvery Hospitals, Radial Road, Chennai, India; 2 Department of Pathology, Kauvery Hospitals, Radial Road, Chennai, India

**Keywords:** Uterine angiomyolipoma, Extrarenal AML, PEComa, Premenopausal woman, Tuberous sclerosis, India

## Abstract

**Background:**

Angiomyolipoma (AML) is a rare benign mesenchymal tumor, originating from perivascular epithelioid cells (PECs), composed of smooth muscle, fat, and thick-walled blood vessels, most often found in the kidney. Uterine AML is exceptionally rare, with under 40 cases reported worldwide and 3 from India prior to the present case. Unlike renal AMLs, uterine lesions are typically sporadic and not linked to tuberous sclerosis (TS), which, combined with their resemblance to fibroids, makes preoperative diagnosis challenging.

**Case Description:**

A 49-year-old woman presented with six months of progressive lower abdominal distension and three months of amenorrhea. She had no signs of TS. Physical examination revealed a firm, non-mobile pelvic mass extending to the xiphisternum. Contrast-enhanced Computed Tomography (CT) showed a 27 × 18 × 19 cm submucosal uterine mass with right hydroureteronephrosis; Cancer Antigen-125 (CA 125 ag) was normal. A total abdominal hysterectomy with bilateral salpingo-oophorectomy was performed, revealing a 7 kg anterior wall tumor. Histopathology confirmed classic AML architecture—interlacing smooth muscle bundles, mature adipocytes, and hyalinized blood vessels—without atypia or mitotic activity. She recovered well.

**Discussion:**

Uterine AML is a “diagnostic chameleon,” often mistaken for leiomyomas, lipoleiomyomas, or degenerated fibroids on imaging. Definitive diagnosis relies on histopathological examination. Each reported case contributes vital insights into this rare gynecologic entity and highlights the need to include AML in the differential diagnosis of unusual uterine masses.

**Conclusion:**

This case underscores the importance of maintaining clinical suspicion for uterine AML when encountering large or atypical uterine lesions. Our patient underwent a hysterectomy and had an uneventful recovery. Histologic confirmation and complete surgical excision are key to optimal management and favorable outcomes, enriching our understanding of extrarenal AMLs in gynecologic practice.

## 1. INTRODUCTION 

Uterine masses are common in gynecological practice, with uterine leiomyomas being the most frequent benign tumors. Affecting up to 70–80% of women by age 50, they may present with abnormal uterine bleeding, pelvic pain, or remain asymptomatic. Although ultrasonography and magnetic resonance imaging (MRI) usually enable a presumptive diagnosis, atypical features in some cases can create diagnostic uncertainty and suggest rare alternative pathologies, including uterine angiomyolipoma, an exceedingly uncommon entity.

Angiomyolipoma (AML) is an uncommon mesenchymal neoplasm derived from perivascular epithelioid cells (PECs), characterized by a distinctive triphasic architecture composed of smooth muscle fibers, mature adipocytes, and blood vessels in varying proportions.^1^ Although AMLs predominantly arise in the kidney and liver, their occurrence within the female reproductive system is comparatively rare, accounting for approximately 0.06% of all benign uterine tumors.^2^ Among gynecologic sites, the uterine corpus is the most frequently affected location, followed sequentially by the cervix, vagina, ovary, fallopian tube, broad ligament, and parametrial tissues.^2–4^

Uterine AML is notably rare, and unlike its renal counterpart, it is not strongly associated with tuberous sclerosis (TS), with reported cases linking only ~7% to the syndrome.^1,2^ Uterine AMLs are consistently benign and most frequently observed in women over 40 years of age.^5^ The patient was managed at a private tertiary care center in India with access to specialized gynecological care and surgical facilities. Written informed consent was obtained from the patient for publication of this case report and the accompanying clinical images.

## 2. CASE PRESENTATION

A 49-year-old premenopausal woman (Gravidity 2; Parity 2; Live 2) of South Asian (Dravidian) ethnicity initially presented herself to our Outpatient Clinic with a 6-month history of progressive lower abdominal distension and a 3-month history of amenorrhea. Prior to that, she had a history of menorrhagia on the first 2 days of her menstrual cycle for a year. The patient had no mass-related pelvic or abdominal pressure symptoms. Apart from hypertension (well controlled with calcium channel blockers), she had no other comorbidities, no features of TS, and no prior surgeries.

Physical examination revealed a non-mobile pelvic mass extending to the xiphisternum. A contrast-enhanced Computed Tomography (CT) scan identified a 27.4 ×18.5x 19.7 cm large heterogeneous enhancing mass originating from the uterine corpus compressing and displacing the endometrium posteriorly, suggestive of a large submucosal leiomyoma/ovarian mass. The uterus itself measured 29.4 x 20.4x 26.4 cm. The right ovarian vein was tortuous and adherent to the mass. Both the ovaries were normal. There was also moderate hydroureteronephrosis. Tumor markers (Cancer Antigen-125 [CA-125]) were within normal limits (9.8U/ml) (Figures 1 and 2).

Following a thorough clinical and radiological assessment, an anesthesia evaluation was done, and she was planned for surgery after two weeks. A multidisciplinary team was involved. In view of difficult bilateral ureteral stenting due to the huge mass, the urologist resorted to double J catheterization, with the letter “J” denoting the J-shaped ends of the tube. During laparotomy, the ovaries and fallopian tubes appeared grossly normal. Macroscopically, a solid mass measuring approximately 29 x 27 cm was noted arising from the anterior uterine wall. Intraoperatively, the diagnosis was leiomyoma uteri. A total hysterectomy with bilateral salpingo-oophorectomy was performed. The bladder was found to be adherent to the mass, and sharp dissection was done. Removal of the huge mass caused sudden hypotension with a systolic blood pressure less than 80mm Hg, and she was started on inotropes (noradrenaline). There was significant blood loss leading to a total administration of three units of packed red blood cells and 2 units of fresh frozen plasma. The mass weighed around 7 kg (Figure 3).

Microscopically, histopathological examination revealed a well-circumscribwed unencapsulated lesion composed of 3 components, predominantly interlacing benign smooth muscle bundles; the lipomatous component was composed of mature adipose tissue, and the angiomatous component was composed of hyalinized blood vessels, consistent with AML. No evidence of mitotic activity, necrosis, or cellular atypia was observed. Hence, a diagnosis of extra-renal AML was made.

The patient was shifted to the intensive care unit (ICU), and inotropes were weaned off gradually. She had an uneventful post-operative period during her hospital stay and was discharged on the third post-operative day with oral hematinic. The patient was followed up in the outpatient clinic at one month and had no complaints or post-operative complications (Figure 4).

## 3. DISCUSSION 

According to the World Health Organization (WHO),^3,6^ perivascular epithelioid cell tumors (PEComas) represent a distinct family of mesenchymal neoplasms characterized by histologically and immunohistochemically unique perivascular epithelioid cells. This tumor group encompasses both renal and extrarenal AMLs, lymphangioleiomyomatosis, and clear cell tumors arising in various anatomical locations.^3,5^

AMLs are further classified into two subtypes: sporadic and syndromic, the latter being associated with the TSC complex (TSC). The sporadic form accounts for approximately 80–90% of AML cases and typically presents in older individuals, independent of any underlying genetic syndrome.^5,7,8^

TS is a rare genetic condition affecting about 1 in 10,000 individuals. It leads to benign tumors, or hamartomas, in multiple organs, including the brain, heart, eyes, skin, bones, kidneys, lungs, and liver. TS is caused by mutations in the TSC1 (chromosome 9q34) or TSC2 (chromosome 16p13) genes, which encode the proteins hamartin and tuberin. These proteins help regulate cell growth. While TS is autosomal dominant, most cases result from spontaneous mutations. Diagnosis relies on clinical signs, imaging, and histopathology due to its varied presentation and systemic involvement.^9^

Differentiating AML from other uterine lesions can be challenging. Lipoleiomyomas, for instance, contain both adipose and smooth muscle tissue but lack the prominent vascular component typical of AML. In contrast, degenerated myomas may also mimic angiomyolipomas; however, they typically present with distinctive imaging features, such as increased echogenicity, absence of acoustic shadowing, and irregular margins, which aid in differentiation.^10^

Grossly, uterine AMLs tend to appear as solitary intramural or subserosal nodules. Their consistency ranges from soft to firm, and the cut surface typically shows a gray to pink-tan hue, reflecting the variable mix of muscle, fat, and vascular elements. No strict quantitative criteria exist for the proportion of each component required to confirm the diagnosis.^5^

A comprehensive review of the literature indicates that uterine AML remains exceedingly rare, with only 36 cases documented worldwide to date; most are reported as isolated case reports.^1,2^ The largest series was published by Wang et al.^1^ comprising eight cases. Mulchandani et al. reported the first case from India in 2020, accompanied by a detailed review of 28 previously published cases.^2^ Subsequently, Pol and Roy described two additional Indian cases in 2022, bringing the total number of Indian reports to three.^11–13^ Our case therefore represents the fourth reported from India. In addition, Laffargue et al.^14^ highlighted a rare occurrence in a 20-year-old primigravida, highlighting the unusual association of uterine AML with pregnancy.

These tumors have been reported across a wide age spectrum, from 20 to 83 years, with a mean age of 48 years. They most commonly arise in the uterine body—particularly the fundus and lower segment—and average around 8 cm in size. Our patient was aged 49, consistent with previously reported patterns, but presented with a 27 cm mass in the anterior uterine wall, indicating the peculiarity and the largest size ever recorded.^2,5^

The clinical presentation of uterine AMLs is generally nonspecific and closely mimics that of leiomyomas. Common symptoms include menometrorrhagia, a palpable pelvic mass, and lower abdominal pain. In our patient, the complication was extreme abdominal distension due to a massive tumor compressing adjacent structures. This contrasts with previously reported cases, where tumors were smaller, and complications were limited to bleeding or pain.^2^

Preoperative evaluation typically involves transvaginal or abdominal ultrasonography, supplemented by CT and MRI. Unlike leiomyomas, uterine AMLs lack classical sonographic features. The presence of a lipomatous component often manifests as increased echogenicity on ultrasound, which can be further characterized by CT or MRI.^2^

Due to their atypical imaging characteristics, many previously reported cases of uterine AML were initially misdiagnosed as teratomas or cystic neoplasms.^2^ In our case, both clinical and radiological assessments erroneously suggested a diagnosis of uterine leiomyoma.

MRI is particularly useful in detecting AMLs, especially when the lesion contains abundant fat, which appears hyperintense on T1- and T2-weighted sequences. This imaging modality enhances sensitivity for identifying extrarenal AMLs.^2^

Immunohistochemistry is instrumental in confirming the diagnosis. Common markers include smooth muscle actin (SMA), desmin, S100, Human Melanoma Black-45 (HMB-45), and Melan-A. HMB-45 positivity in nonvascular smooth muscle cells is a hallmark of AMLs in organs like the kidney and colon, though it is less frequently observed in uterine cases.^14–16^

In the present case, no immunohistochemical staining was performed, and the diagnosis was established solely on the basis of histopathological examination, which revealed the classic triphasic morphology of AML.

Surgical excision remains the mainstay of treatment, with most cases managed via total abdominal hysterectomy, sometimes accompanied by bilateral salpingo-oophorectomy depending on age and clinical context.^1^ While this approach is widely adopted, Cil et al. have proposed that active surveillance may be appropriate for patients presenting with smaller, asymptomatic lesions, thereby avoiding unnecessary surgical intervention. Our patient underwent hysterectomy due to its massive size. Prognosis following complete resection is generally excellent, with minimal risk of recurrence. Nonetheless, regular follow-up is recommended, particularly for patients managed non-surgically.

## 4. CONCLUSION

Uterine AML is exceptionally rare. Our patient presented with nonspecific clinical features that closely mimicked leiomyoma, underscoring the diagnostic challenges associated with this tumor. Preoperative imaging raised suspicion, but definitive diagnosis was established only after histopathological examination. The patient underwent successful surgical excision of the mass, which weighed around 7 kg, representing the largest uterine AML reported to date. Postoperatively, she recovered well, highlighting that timely recognition and appropriate surgical management can lead to favorable outcomes even in such rare and unusually large presentations.

## ACKNOWLEDGEMENT

The author would like to thank Dr Raghavi Nanthakumar, Registrar, Department of Pathology, Clinical Laboratory, Kauvery Hospitals, Radial Road, Chennai, India, and Mr Dinesh Kumar, In-charge, Clinical Laboratory, Kauvery Hospitals, Radial Road, Chennai, India.

## AUTHORS’ CONTRIBUTIONS

All authors have contributed to the study’s conceptualization and design. SM, TK, and AS. SM, TK, AS, and FM: Material preparation, data collection, and analysis. AS: Writing original draft. SM, TK, AS, and FM: Writing and review. All authors read and approved the final manuscript.

## CONFLICT OF INTEREST

The authors declare that they have no conflict of interest.

## DISCLOSURE OF AI USE

The authors declare that no artificial intelligence (AI) or AI-assisted technologies were used in the creation, drafting, or editing of this manuscript.

## DATA AVAILABILITY

The data associated with the paper are not publicly available but are available from the corresponding author upon reasonable request.

## FUNDING

The author(s) received no financial support for the research, authorship, and/or publication of this article.

## ETHICAL APPROVAL

Ethical approval was waived by the local Ethics Committee of our institution in view of the retrospective nature of the study, and all the procedures being performed were part of the routine care.

## PATIENT CONSENT

The authors certify that they have obtained the appropriate patient consent forms wherein the patient has consented for her images and other clinical information to be reported in the journal. The patient understands that her name and initials will not be published, and due efforts will be made to conceal her identity.

## Figures and Tables

**Figure 1.**
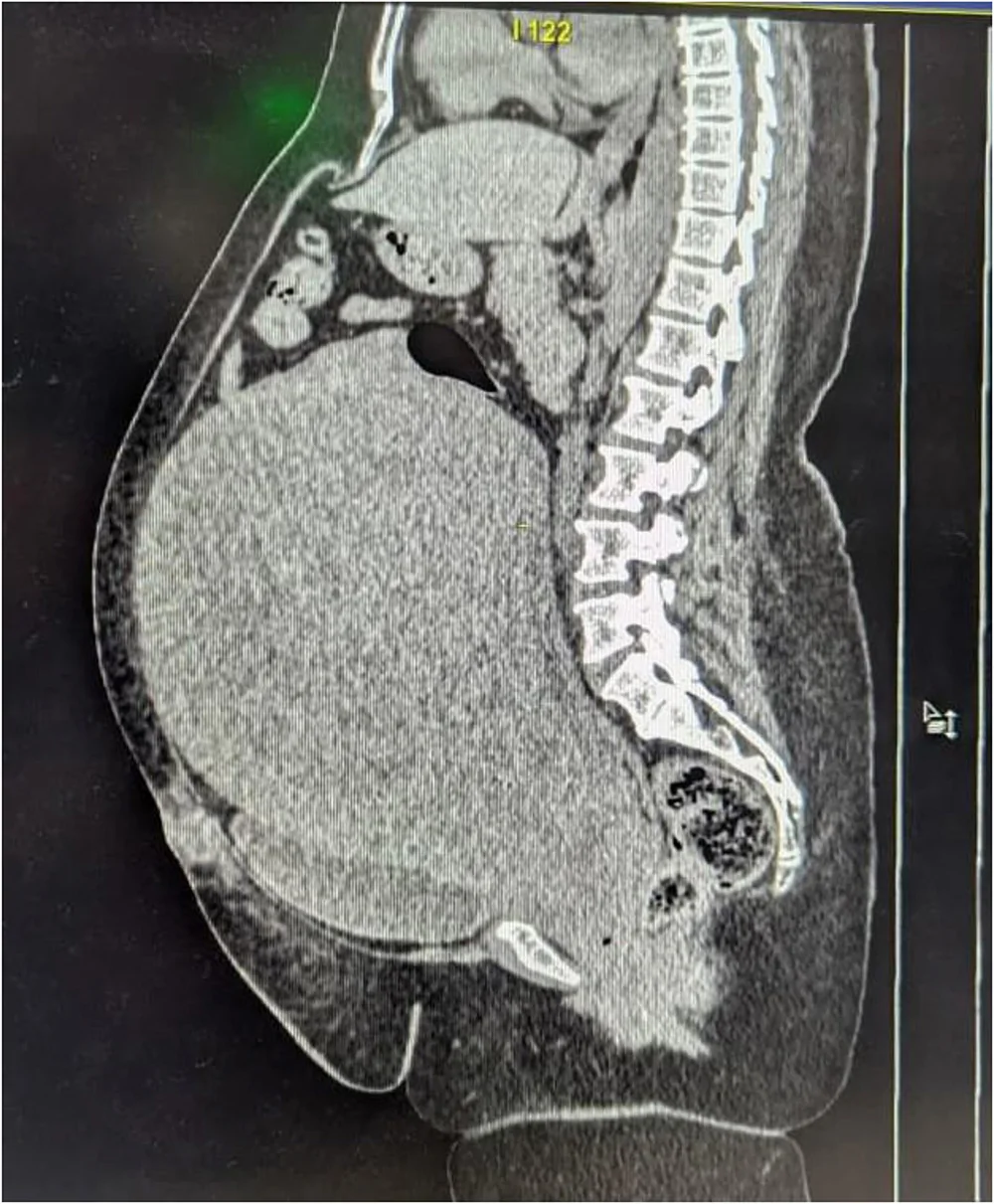
Sagittal view of contrast-enhanced CT image showing a large heterogeneous mass arising from the uterus, compressing and displacing the endometrium posteriorly.

**Figure 2.**
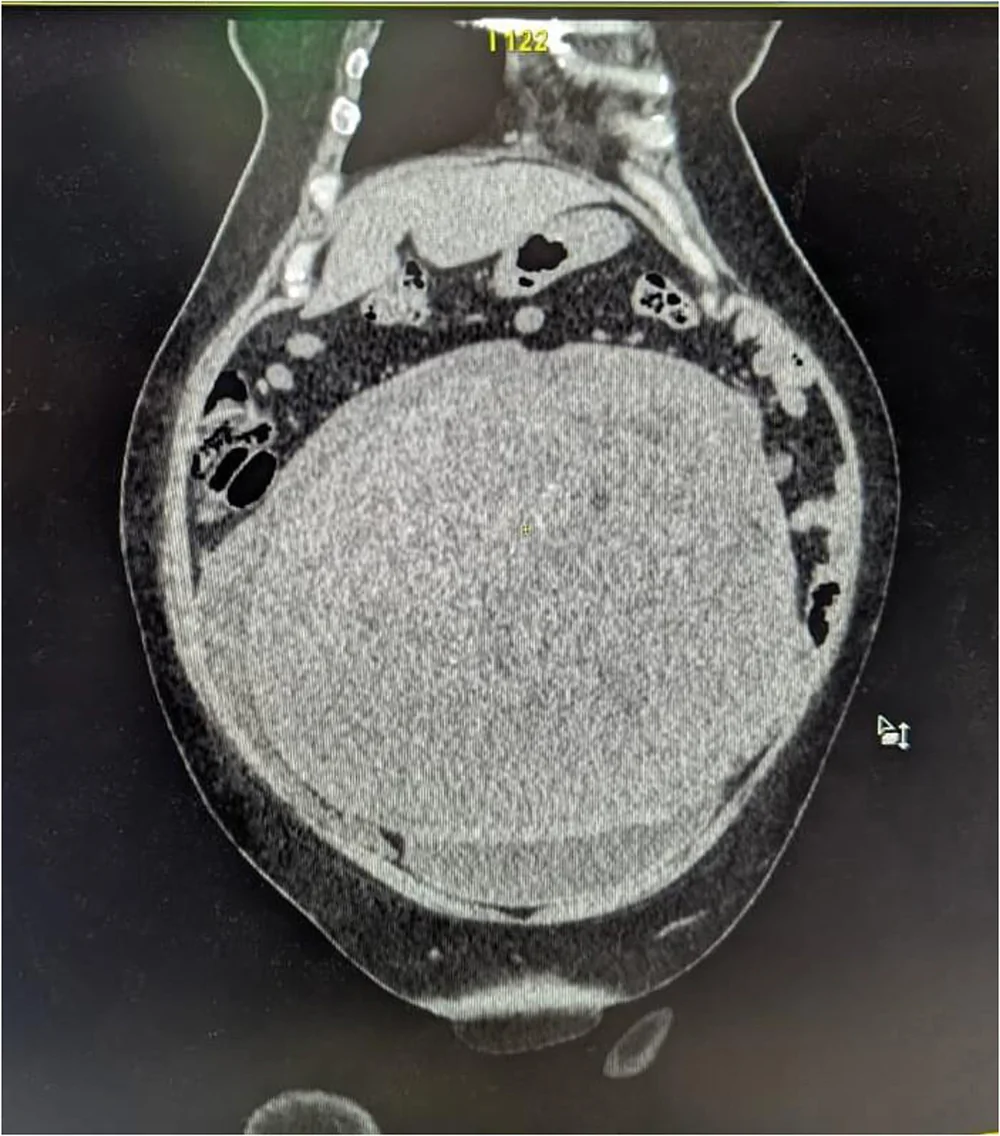
Axial view of contrast-enhanced CT image showing the uterus occupying the entire abdomen and pelvis.

**Figure 3.**
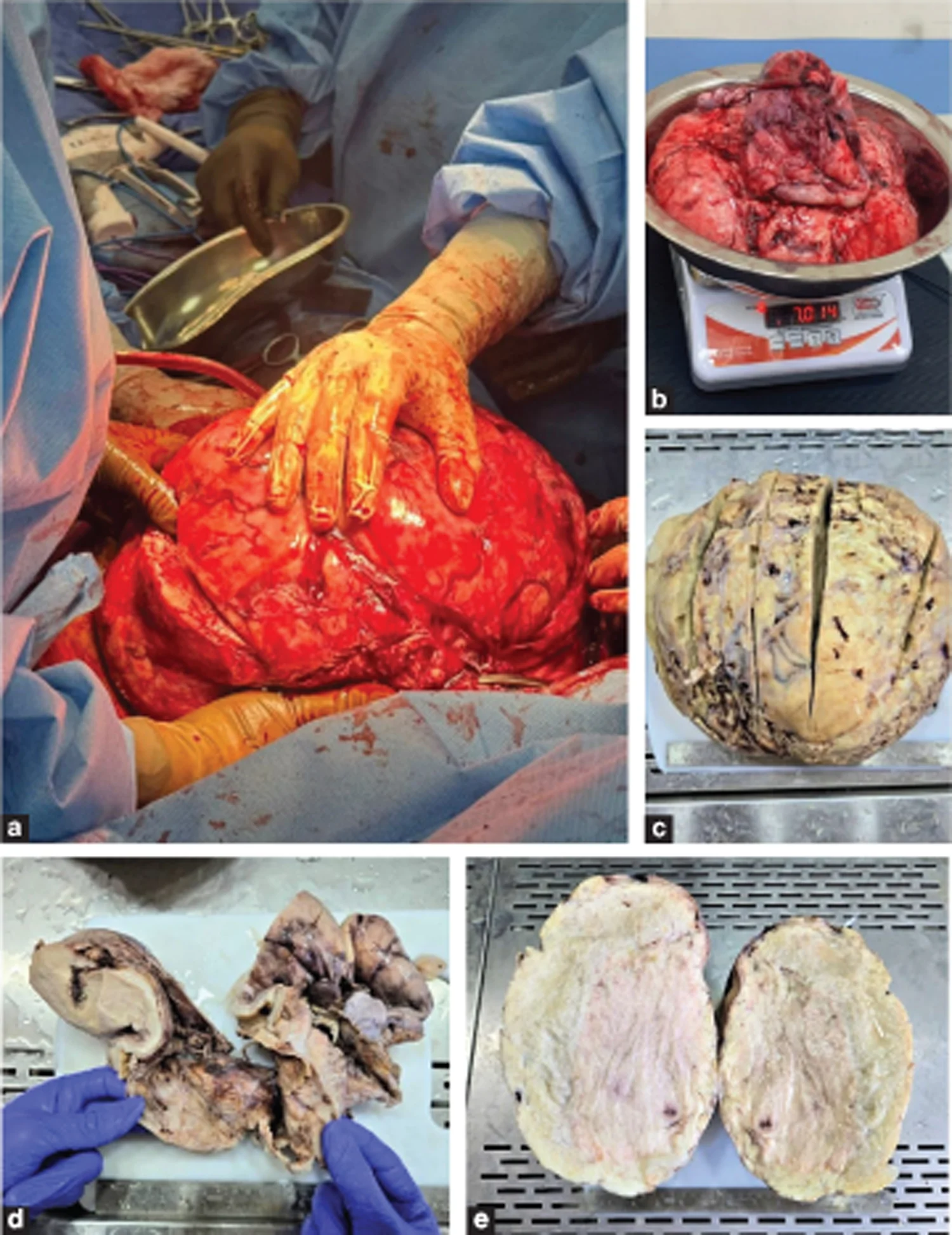
Intra-operative view of the mass (a); excised tumor weighing 7.014 kg (b); tumor mass removed from the anterior wall of the uterus (c); cavity in the uterine wall following excision (d); and cut surface of the tumor (e), which appears yellowish white in color and firm in consistency.

**Figure 4.**
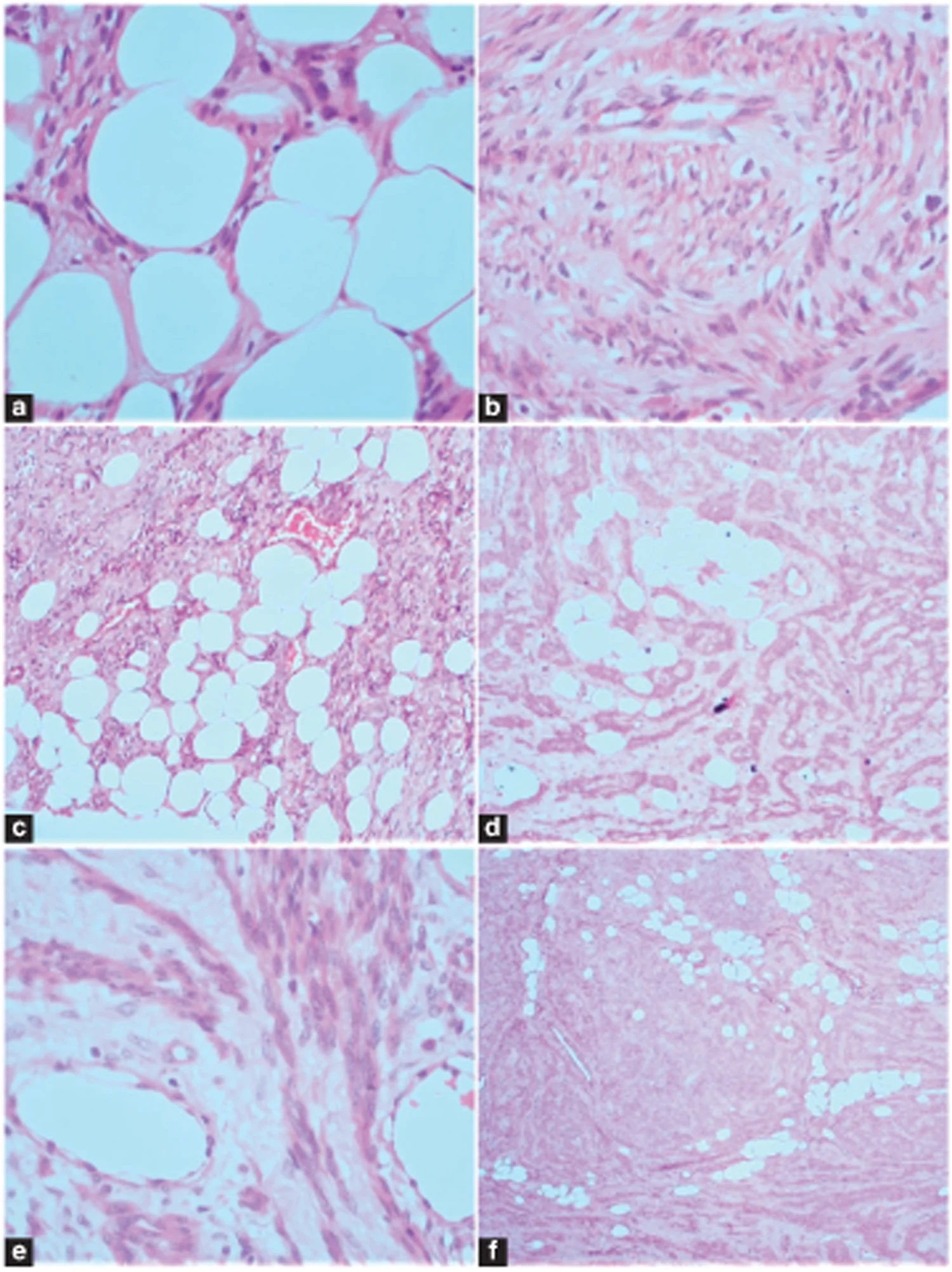
Photomicrograph of the lipomatous component of the tumor (a); photomicrograph of the smooth muscle component of the tumor with whorled fibers (b); photomicrograph of the lipomatous component showing adipose tissue surrounded by smooth muscle fibers (c); microphotograph of the tumor demonstrating all three components, including smooth muscle tissue (Hematoxylin & Eosin, ×100) (d); photomicrograph of the angiomatous component with hyalinized blood vessel walls (e); microphotograph of the tumor showing a mixture of the three components (Hematoxylin & Eosin, ×40) (f).
